# Primary headache management in a multidisciplinary team – a pilot study

**DOI:** 10.25122/jml-2023-0259

**Published:** 2023-07

**Authors:** Judit Mihaiu, Daiana Debucean, Petru Mihancea, Adrian Marius Maghiar, Olivia Andreea Marcu

**Affiliations:** 1 Doctoral School of Biomedical Sciences, University of Oradea, Oradea, Romania; 2 Faculty of Medicine and Pharmacy, University of Oradea, Oradea, Romania

**Keywords:** tension-type headache, migraine, bruxism, manual therapy, counseling, TTH–tension-type headache, SB–sleep bruxism, AB–awake bruxism, MT–manual therapy, C–counseling, GP–general practitioner, COP–cervicogenic orofacial pain, TTH ps–tension-type headache pain score, M ps–migraine pain score, LJO–limited jaw opening, Anx–anxiety, PS–perceived stress, CM–chronic migraine

## Abstract

Tension-type headaches and migraines are bidirectionally related to dysfunctions of the cervical and masticatory muscles and some psychosocial factors. Our pilot study aimed to investigate the connections between primary headaches, bruxism, and psychosocial issues. In addition, we aimed to assess the effectiveness of a multidisciplinary approach in decreasing the intensity and frequency of headache episodes and bruxism. Sixty-seven patients previously diagnosed with primary headache and bruxism were divided into two similar groups. One group benefited from manual therapy alone, while the other received manual therapy and counseling sessions for three months. Statistical data analysis was conducted using SPSS, Version 24, using the paired Student’s t-tests and McNemar’s tests. After the three-month intervention period, we observed substantial improvements across various parameters. Some demonstrated statistically significant differences, while others did not reach statistical significance. When comparing the outcomes, the combined therapy proved more effective than manual therapy alone.

## INTRODUCTION

Humans experience headaches more frequently than any other type of pain. These have been described, classified, and treated since antiquity but never fully known and clarified. Tension-type headaches (TTH) and migraines are the most prevalent primary headache disorders in the general population [1-3]. Although all forms of headaches affect the quality of daily life, migraines have the strongest impact, especially on young adults [4, 5]. The etiology of primary headache is diverse and often multifactorial, including family history, gender, age, the general state of the body, and lifestyle, but also causes related to the cervical and facial area [6].

The most significant symptom of TTH is increased interictal pericranial sensitivity (recorded by manual palpation), typically exacerbated during the crisis [7]. A local sensitivity is observed in the frontal, temporal, masseter, pterygoid, sternocleidomastoid, splenius, and trapezius muscles. The precise mechanism is not fully known, but peripheral and central pain mechanisms are considered [8, 9]. Moreover, the connections with the emotional state cannot be neglected in TTH. Patients with depression or anxiety often perceive pain as much more intense and disabling than the general population [10].

Unlike TTH, migraines occur only on one side of the head (usually felt behind the eye) and exhibit a pulsating character that gradually worsens. Genetics are often identified as the primary factor contributing to migraines [11].

The available literature indicates connections between the high prevalence of tension-type headaches (TTH) and migraines and the dysfunction of specific muscle groups, specifically the cervical and masticatory muscles [12]. Additionally, there are associations with certain psychosocial factors, including stress, anxiety, and depression, which may manifest behaviorally as bruxism [13, 14].

Bruxism is considered a behavior of the masticatory muscles [15] and is a generic term that encompasses two distinct entities: sleep bruxism and awake bruxism. Sleep bruxism (SB) occurs during phases of shallow sleep in an estimated 13% of the population. Awake bruxism (AB) is a state of permanent dental contact (clenching) and/or contraction of the masticatory muscles, ranging from 22.1% to 31% in the general population [16], with a higher occurrence among women [17]. While it is typically considered a repetitive parafunction [18, 19], bruxism can also have some positive consequences, leading to a distinction between “normo-bruxism” and “patho-bruxism” [20]. Patho-bruxism is associated with adverse effects such as tooth wear, temporomandibular disorders, and muscle pain. Diagnosis for both types relies on self-observation, electromyography, and polysomnography. The etiology is most likely multifactorial. The idea of occlusal origin has been abandoned recently, as correlations between bruxism and anatomical-structural factors could not be established [21]. The literature claims that SB has central, physiopathological (level of brain activation, level of neurotransmitters, genetic factors, smoking, alcohol or drug consumption, mineral deficiencies, allergies) and psychosocial origins (stress, anxiety, personality type, low social support), the latter also being at the origin of AB [22].

The association between bruxism and primary headache has been intensely debated, and the consensus is towards a significant association between TTH, migraine, and sleep bruxism [23, 24]. In our clinical practice, we have observed that patients suffering from headaches often exhibit anxiety symptoms and increased stress. Furthermore, their medical histories have shown a higher frequency of bruxism. Given that we typically treat headaches with manual therapy and address emotional issues through counseling, we aimed to assess whether combining these two approaches could increase the quality of the results obtained. This construct is supported by existing literature highlighting the connections between primary headaches, bruxism, and psychosocial factors [25, 26].

This study aimed to evaluate the effectiveness of a combined, multidisciplinary approach involving manual therapy (MT) and counseling (C) in reducing headache intensity, anxiety levels, perceived stress, and the incidence of bruxism when compared to manual therapy (MT) alone.

## MATERIAL AND METHODS

### Study design and participants

This randomized trial was conducted throughout 2021 and took place in two private practice offices: a physiotherapy–manual therapy office and a psychology-counseling office. A total of 67 patients, 9 men and 58 women aged 12-64, participated in the study. They were all diagnosed with primary headaches (TTH, migraine, or both), and 58 also had bruxism (AB, SB, or both) and high levels of anxiety and perceived stress. Most participants received a prior medical diagnosis from a general practitioner (GP) or neurologist for their primary headaches, while anxiety was diagnosed in 9 patients initially by a GP and subsequently confirmed by a psychologist. Participants and parents of minor participants provided written informed consent before inclusion in the study. The participants and the parents of the minors were also informed that they could withdraw at any moment during the study without consequences.

### Group allocation

Participants were divided into 2 groups with almost the same number of subjects (33 versus 34 subjects), considering the patient’s motivation to receive manual therapy and in-home exercises but also psychological counseling and in-home relaxation techniques. The inclusion criteria in the study comprised subjects with a history of TTH and/or migraine and at least one of the following: bruxism, anxiety, stress, and consent to participate. Although bruxism, anxiety, and high levels of anxiety were present in almost all our patients, these occurred in variable combinations. The exclusion criteria were refusal to participate, subjects attending other rehabilitation procedures or psychotherapy, and chronic medication prescribed (analgesic or psychiatric drugs).

### Clinical assessment

While most participants had a medical diagnosis of primary headache, a thorough functional assessment was conducted by the physiotherapist, including a comprehensive anamnesis. This assessment was based on observations, patient reports, questionnaires, and specific tests, all aligned with The International Classification of Headache Disorders - 3rd edition (ICHD-3). The anamnesis questions referred to the location and the characteristics of the pain, the duration and frequency of the episodes, their periodicity and evolution over time, triggers, known factors that alleviate or worsen the pain, and other symptoms that occur before, during, and after a headache episode.

TTH was characterized by bilaterally localized pressing or constricting pain (but not pulsating) of mild or moderate intensity, with a duration of up to one week, with an average of 4 to 6 hours [7]. ICHD-3 distinguishes between infrequent or frequent episodic and chronic TTH. All patients had an increased pericranial tenderness detected by manual palpation, as well as parameter intensity tenderness in frontal, temporal, masseter, pterygoid, sternocleidomastoid, splenius, upper fasciculus of the trapezius and the suboccipital muscles. These were also the muscular groups to which the manual therapy techniques were applied. The pain was not increased by everyday activity.

Migraine diagnosis was based on the ICHD-3 criteria, including a headache duration of 4 to 72 hours, the presence of two or more characteristics such as unilateral location, pulsating, and moderate to severe pain usually worsened by everyday activities (climbing stairs, walking), and accompanying symptoms (more than one of them): nausea, vomiting, photo- and/or phonophobia, dizziness. All these symptoms are distinctive features of the migraine and differentiate it from other headaches. Sometimes, it is difficult to differentiate between TTH and migraine because they may overlap in the same person – in 25 of our patients (3 men and 22 women).

A differential diagnosis must be made between migraine and cluster headache; the latter has severe to very severe intensity, occurs typically around or behind one eye, lasts 15 minutes to 3 hours, and may be accompanied by nasal congestion, eyelid drooping or edema, eye redness, tearing, restlessness. Also, headaches affect men three times more frequently than women when compared to migraines and TTH [7]. Another differential diagnosis must be made between migraine and cervicogenic orofacial pain (COP). While the pain in COP radiates in a posterior-anterior direction, in migraine, it is anterior-posterior [27].

### Diagnostic instruments

The intensity of pain was measured using the Visual Analogue Scale. The subjects marked their perceived pain level between “no pain at all” and “pain as bad as it could be”. To evaluate jaw mobility limitations, we employed the Jaw Functional Limitation Scale (JFLS) [28], a comprehensive assessment tool consisting of 20 items. The JFLS assesses various aspects, including mastication, vertical jaw mobility, as well as verbal and emotional expression. This instrument has demonstrated high reliability and validity, with a Cronbach’s alpha of 0.87 and 0.87 temporal stability, ideal for clinical practice and research [29].

The assessment of bruxism was self-reported, the abnormal tooth wear observed, and the morning pain and fatigue of the facial muscles [30]. The BruxApp^®^, a dedicated smartphone application for reporting awake bruxism, was used to assess AB. It reports some oral conditions linked to AB: state of the jaw muscles, presence of tooth contact, clenching, grinding, and mandible bracing [31]. Data were recorded 4 times a day for 7 days, twice (at the beginning and the end of the study).

Anxiety and stress are normal parts of our lives when experiencing them occasionally, but when they are intense, persistent and excessive, they greatly impact everyday situations. The Diagnostic and Statistical Manual of Mental Disorders (DSM-5) [32] defines anxiety as an excessive worry that affects everyday life, often accompanied by muscle tension. In order to assess the emotional status of the patients, we used informal and formal evaluation: clinical interviewing and the Depression, Anxiety, Stress Scale (DASS-21R) [33]. We aimed to assess the levels of anxiety and stress and not set up a diagnosis of mental disorder or treat it specifically. These can be later subjects of psychotherapy, where needed. When these scores were too high, meaning they had clinical intensity and significance, the patients were referred to a psychiatrist.

The clinical interviewing comprised non-judgmental open-ended questions, non-directive listening, talking, confrontation, and interpretation. We used the interviewing to set up a safe environment where the patient could discuss worries, fears, feelings, and distressing symptoms collaboratively.

DASS-21R is a 21-item questionnaire with good, reliable psychometric properties (total Cronbach's alpha between 0.89 - 0.93) [34] that assesses negative emotional states. Symptoms of anxiety and changes in the sleep pattern were correlated with headaches.

### Intervention

The rehabilitation plan included two groups:

**Manual therapy (MT) group:** Participants attended MT sessions twice a week, along with daily in-home exercises. The MT sessions included trigger point treatment, soft tissue mobilization, elongation, muscle release, stretching, cranio-cervical muscle exercises, suboccipital inhibition, occiput-atlas-axis mobilization, and head and neck massage. The in-home exercises were designed for posture improvement and stretching. The techniques and exercises were only partly the same for all the patients, and the plans were personalized according to the complaints of each patient and the assessment performed.

**MT+Counseling (MT+C) group:** Patients in this group received MT twice a week, combined with counseling sessions once a week, as well as daily exercises and relaxation techniques at home. The counseling sessions focused on sleep hygiene, a healthy lifestyle, problem-solving, and decision-making techniques. The relaxation techniques suggested for the home included breathing exercises (learned in the sessions and then practiced at home, e.g., 4-7-8 technique) and grounding exercises.

The MT and the in-home exercises were performed and supervised by a physiotherapist. The counseling sessions and the relaxation techniques and exercises were conducted and supervised by a psychologist. After three months, the patients underwent a new assessment with the same instruments and continued their personalized treatment. The outcomes assessed were pain, anxiety, and perceived stress scores, as well as the awake bruxism report.

### Statistical analysis

The statistical analysis was performed using the Statistical Package for Social Sciences (SPSS), Version 24. The design of the study focused mainly on qualitative parameters, such as awake bruxism (AB), TTH pain score (TTH-ps), migraine pain score (M-ps), limited jaw opening (LJO), anxiety (ANX) and perceived stress (PS), age and SB as quantitative parameter. To evaluate changes before and after therapy, quantitative parameters were analyzed using the paired Student’s t-test. Qualitative parameters were assessed using McNemar's test. Furthermore, to determine differences among the procedures after the three-month interval, Student’s t-test for independent samples was employed for quantitative parameters, while the Chi-Square test was applied for qualitative parameters. A significance level of 0.05 was considered, otherwise mentioned.

## RESULTS

The study involved 67 participants, 58 women and 9 men, aged 12 to 64 years. Women ranged from 12 to 64 years, with an average age of 34.31. Men had ages ranging from 29 to 63 years, with an average age of 37.66 years. Out of the total study population, 9 participants (13.5%) were male, while 58 participants (86.5%) were female, with an average age of 34 and a mean deviation from the average age of 10.83 years. Regarding the overall prevalence of headache, there were 58 women *vs*. 9 men, in general; only TTH: 34 women *vs*. 6 men; only migraine 2 women *vs*. 0. Furthermore, 25 patients reported experiencing both TTH and migraine concurrently.

We analyzed SB as a quantitative parameter and qualitative parameters, including awake bruxism (AB), TTH pain score (TTH-ps), migraine pain score (M-ps), limited jaw opening (LJO), anxiety (ANX) and perceived stress (PS). The differences in the qualitative parameters between the two groups at the time of assessment were analyzed using Student’s t-test analyses, as presented in Table 1.

**Table 1:** Baseline descriptive characteristics of qualitative parameters

Variable	MT	MT+C	p-value
	Mean±SD	Mean±SD	
Age	35.3824±13.01052	33.303±8.19102	0.436
AB	104.5±17.438	110.41±13.616	0.25
TTH Ps	8.0882±0.96508	7.7091±0.82436	0.096*
M ps	8.64±0.929	8.54±0.877	0.767
LJO	98.2857±24.73064	101.2727±26.59733	0.774
Anx	16.8235±5.70205	16.1818±6.29213	0.663
PS	24.7353±7.11511	25.1515±7.11511	0.807

* significant at 0.1

MT=manual therapy; MT+C=manual therapy and counseling; AB=awake bruxism; TTH ps=tension-type headache pain score; M ps=migraine pain score; LJO=limited jaw opening; Anx=anxiety; PS=perceived stress

Among the parameters assessed, awake bruxism and perceived stress had statistically significant differences. AB had an average score of 85.67 (±18.11) in the MT group, whereas it averaged 74.53 (±14.067) in the MT+C group. The mean PS in the MT group was 19.9412 (±5.29117) and 17.5758 (±4.2649) in the MT+C group. Additionally, the average age in the MT group was 35.38 (±13.01), while in the MT+C group, it was 33.30 (±8.19). The TTH-ps presents significant differences if a level of significance of 0.1 is considered.

To evaluate the changes before and after therapy, we conducted a paired Student’s t-test for quantitative parameters (Table 2) and employed McNemar’s test for qualitative variables. We also assessed the differences among the procedures after the three-month interval using Student’s t-test for independent samples and Chi-square test. A significance level of 0.05 was considered.

**Table 2:** Prevalence of SB before and after the procedures

Variable	Before procedure n(%)	After procedure n(%)	p-value
SB	58 (86.5%)	51 (76.1%)	0.12

* SB=sleep bruxism

As seen in Table 2, 58 (86.5%) out of the total study population confirmed sleep bruxism at the initial time, with an improvement to 51 (76.1%) after the 3 months. The improvement in the number of patients confirming sleep bruxism was not statistically significant. The descriptive characteristics of the qualitative parameters before and after the three-month interval are presented in Table 3.

**Table 3:** Descriptive characteristics of qualitative parameters before and after procedures

Variable	Before	After	p-value
	Mean±SD	Mean±SD	
AB	106.9512±16.05296	81.0488±17.27998	0
TTH ps	7.9077±0.91384	4±1.0155	0
M ps	8.59±0.888	4.6667±1.07417	0
LJO	99.6±25.06658	45.04±17.84302	0.001
Anx	16.5075±5.96295	13.0448±4.34299	0
PS	24.9403±6.88626	18 .7761±4.92311	0

AB=awake bruxism; TTH ps=tension-type headache pain score; M ps=migraine pain score; LJO=limited jaw opening; Anx=anxiety; PS=perceived stress

**Figure 1:**
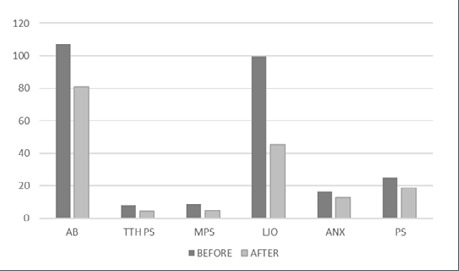
Mean value comparisons before and after any procedure AB=awake bruxism; TTH ps=tension-type headache pain score; M ps=migraine pain score; LJO=limited jaw opening; Anx=anxiety; PS=perceived stress

All qualitative parameters decreased significantly after 3 months, as seen in Figure 1. The mean score of AB improved from an average of 106.9512 (±16.05296) to 81.0488 (±17.27998). The mean score for TTH-ps improved significantly from 7.9077 (±0.91384) to 4 (±1.0155). The mean pain score for migraine decreased from 8.59 (±0.888) to 4.6667 (±1.07417). Limited jaw opening mean score improved from 99.6 (±25.06658) to 45.04 (±17.84302). On average, anxiety level decreased from 16.5075 (±5.96295) to 13.0448 (±4.34299), and the mean perceived stress level improved from 24.9403 (±6.88626) to 18.7761(±4.92311).

Over the course of three months, participants underwent rehabilitative procedures, with one group receiving manual therapy alone and the other receiving a combination of manual therapy and counseling. Table 4 provides descriptive statistics (gender and sleep bruxism) for the two patient groups, MT compared to MT+C, at the time of assessment.

**Table 4:** Baseline descriptive characteristics for gender and SB

Variable		All patients	MT	MT+C	p-value
		n (%)	n (%)	n (%)	
sex	F	58 (86.56%)	29 (85.29%)	29 (87.87%)	0.756
	M	9 (13.43%)	5 (14.71%)	4 (12.13%)	
SB	yes	58 (86.56%)	30 (88.23%)	28 (84.84%)	0.684

MT=manual therapy; MT+C=manual therapy and counselling; SB=sleep bruxism

Among the total of 67 patients, 58 (86.56%) were women, and 9 (13.43%) were men. Of these, 29 women and 5 men received the MT procedure, while 29 men and 4 women underwent the MT+C treatment. There were no significant differences in the distribution of gender and sleep bruxism between the two types of procedures. The descriptive characteristics for SB after 3 months are presented in Table 5, comparing the two groups.

**Table 5:** Descriptive characteristics of SB after 3 months

Variable		All patients	MT	MT+C	p-value
		n (%)	n (%)	n (%)	
SB	yes	51 (76.1%)	26 (76.5%)	25 (75.7%)	0.945

SB=sleep bruxism; MT=manual therapy; MT+C=manual therapy and counseling

51 (76.1%) patients confirmed sleep bruxism, out of which 26 (76.5%) underwent MT only and 25 (75.7%) MT+C. There were no significant differences in the sleep bruxism distribution related to the type of treatment. Table 6 illustrates the variations in outcomes achieved with each treatment approach over the three months.

**Table 6:** Differences in treatment outcomes at 3 months

Variable	MT	MT+C	p-value
	Mean±SD	Mean±SD	
Age	35.3824±13.01052	33.303±8.19102	0.436
AB	85.67±18.11	74.53±14.067	0.04
TTH ps	4.0882±0.99598	3.9032±1.04419	0.468
M ps	4.79±0.802	4.54±1.33	0.569
LJO	48.4286±21.9955	40.7273±9.93067	0.258
Anx	13.7647±4.31389	12.303±4.31194	0.17
Ps	19.9412±5.29117	17.5758±4.2649	0.048

MT=manual therapy; MT+C=manual therapy and counseling; AB=awake bruxism; TTH ps=tension-type headache pain score; M ps=migraine pain score; LJO=limited jaw opening; Anx=anxiety; PS=perceived stress

The only parameters significantly different were AB and PS. AB had an average score of 85.67 (±18.11) in the MT group and 74.53(±14.067) in the MT+C group. PS had an average score of 19.9412 (±5.29117) in the MT group and 17.5758(±4.2649) in the MT+C group. The average age in the MT group was 35.3824 (±13.01052), and 33.303(±8.19102) in the MT+C group. Table 7 shows the SB comparison within the MT group at two time points: the initial assessment and the three-month follow-up.

**Table 7:** SB in the MT group, before and after manual therapy

Variable	Before n (%)	After n (%)	p-value
SB	30 (88.23%)	26 (76.47%)	0.17

SB=sleep bruxism

No significant differences were observed between the distribution of SB at the time of assessment and after 3 months in the group of subjects treated only with manual therapy. 30 (88.23%) patients reported sleep bruxism at the initial assessment, and after 3 months, only 26 (76.47%) still reported it. The results for the qualitative parameters are presented in Table 8.

**Table 8:** Qualitative parameter results before and after three months of MT

Variable	Before	After	p-value
	Mean±SD	Mean±SD	
AB	104.5±17.438	85.6667±18.10997	0
TTH ps	8.0882±0.96508	4.0882±0.99598	0
M ps	8.64±0.929	4.7857±0.80178	0
LJO	98.2857±24.73064	48.2486±21.9955	0
M ps	16.8235±5.70205	13.7647±4.31389	0
PS	24.7353±7.11511	19.9412±5.29117	0

AB=awake bruxism; TTH ps=tension-type headache pain score; M ps=migraine pain score; LJO=limited jaw opening; Anx=anxiety; PS=perceived stress

All the qualitative parameters were significantly different after 3 months of manual therapy. As for the MT+C group, Table 9 presents the descriptive statistics for baseline SB and after 3 months of follow-up.

**Table 9:** SB in the MT+C group, before and after therapies

Variable	Before n(%)	After n(%)	p-value
SB	28 (84.84%)	25 (75.75%)	0.269

SB=sleep bruxism

No significant differences were seen in the distribution of SB between baseline assessment (28 patients, 84.84%) and after the 3 months (25 patients, 75.75%). The differences between the qualitative parameters measured before and after the MT+C are presented in Table 10.

**Table 10:** Qualitative parameter results before and after MT-C

Variable	Before	After	p-value
	Mean±SD	Mean±SD	
AB	110.41±13.616	74.5294±14.06733	0
TTH ps	7.7097±0.82436	3.9032±1.04419	0
M ps	8.54±0.877	4.5385±1.33012	0
LJO	101.2727±26.59733	40.7273±9.93067	0
Anx	16.1818±6.29213	12.3030±4.31194	0
PS	25.1515±6.74593	17.5758±4.2649	0

AB=awake bruxism; TTH ps=tension-type headache pain score; M ps=migraine pain score; LJO=limited jaw opening; Anx=anxiety; PS=perceived stress

The differences between the two moments (baseline assessment and after 3 months) were significant for all parameters.

## DISCUSSION

Headache disorders exhibit a higher prevalence among women. According to data from the World Health Organization, TTH affects roughly three women for every two men, and the ratio for migraine is approximately two women to every one man, partly due to hormonal factors. The gender distribution in our study sample may be a subject of discussion since the women-men ratio was highly distorted. One possible explanation for this gender disparity could be that men may be less inclined to explore or consider alternative or complementary treatment methods [35].

Hands-on manual therapy was used to pinpoint tight or stiff muscles and trigger points and create a release, which leads to pain release as well. Elongations, mild activation or deep friction of some specific muscles, suboccipital soft tissue inhibition, and myofascial trigger point therapy [36] allow the soft and connection tissues to relax and eventually realign to release pain. They work best in the cases of tension-type headaches [37]. On the other hand, manual therapy helps with migraines that worsen because of associated stiff muscles and joints, but they are mostly compensatory measures, relieving the muscular adaptations that increase pain [38, 39]. Even so, it is a risk-free approach and may be worth considering as a treatment option.

Different MT and pain reduction methods involve biomechanical, neurophysiological, and psychological components. The philosophy behind these methods is that MT may activate the descending inhibitory pathways via different levels of the spinal cord, so it may be effective in treating CM [36].

Other approaches used were mobilizations, cranio-cervical muscle exercises, occiput-atlas-axis mobilizations, and posture correction exercises, some also in-home. At each session, the manual therapist applied a personalized set of techniques. The manual therapy interventions used were based on the assumption that they decreased sensitization, as described in another study [40].

Data showed a strong association between bruxism (either awake or sleep bruxism) and tension-type headache. Only 8 out of the 67 subjects reported the absence of any type of bruxism, while all the others reported SB. Seventeen subjects reported not experiencing AB. Literature shows a straight relationship between the frequency of morning headaches and sleep bruxism, especially in adults [41]. Patients who experience sleep bruxism report three times as many episodes of headache compared to those without bruxism [42], indicating a potential link between inadequate sleep and headache occurrence. The temporomandibular joint disorders increase the risks of migraines or tension-type headaches [23].

AB seems to have a significant relationship with tension-type headaches and migraines. An explanation would be that the persistent diurnal clenching maintains the tension of the masticatory muscles at a high level so that, over time, there is an increased sensitization of the nociceptors of the peripheral muscles and a change in the stimulus-response function [43].

AB is considered a jaw-clenching habit accompanying stress and anxiety [44], while SB is a sleep-related masticatory activity generally associated with arousal [45].

The association between sleep bruxism and migraine episodes is unclear, although some studies show correlations [46]. There is no current specific treatment to stop SB. Treatments based on behavior changes - relaxation techniques, habit awareness, habit reversal therapy, and sleep hygiene- may eliminate awake bruxism [47]. Sleep hygiene techniques (e.g., relaxation exercises, avoiding alcohol, caffeine, and large meals before sleeping), relaxation and anxiety control techniques are recommended to control sleep bruxism [48], although their efficacity on muscular activity control is debatable [49].

There was a slight difference, without statistical significance, between the two groups regarding the improvement of SB occurrence: only 4 patients in the MT group and 3 in the MT+C group reported it. Since both groups benefited from MT, this variation could be attributed to factors such as the duration of the condition before treatment (with longer-lasting symptoms possibly showing less improvement), unexplored psychological variables in the study (such as depression), or individual personality traits.

At the moment of assessment, no significant differences were noticed in any of the parameters between the two groups. After the three months and the procedures, all the parameters had decreased mean values. Even if the difference was not statistically significant, we noticed better outcomes in the MT+C group, especially for AB, PS, and LJO. The greater improvement in jaw opening may be due to the motivational-affective and cognitive-evaluative nature of pain – several unpleasant emotional experiences lead to fear of pain, the certainty of future pain, and avoidance behaviors that reinforce the movement limitation. All patients reporting LJO also reported SB and AB. Studies confirm a linear relationship between morning headaches and mean anxiety scores [41].

The follow-up assessment after three months demonstrates the efficacy of manual therapy, whether administered alone or in combination with counseling and stress management techniques, in mitigating the intensity, frequency, and overall quality of life for patients dealing with TTH and migraine.

While the results may be modest, they hold promise, especially considering the prevalence of headaches and the limitations of drug therapy in certain population groups, including children, pregnant women, and individuals with specific medical conditions or comorbidities. The MT and MT+C sessions positively influenced parameters related to disability caused by TTH and in everyday life quality improvement. Additionally, MT contributed to a reduction in the local pain threshold. The results of the study underline, in agreement with other studies [50-53], the connection between headaches, bruxism, and psycho-emotional issues. The outcomes of the applied therapies demonstrated that the multidisciplinary approach is superior to the single therapy approach, addressing the underlying symptoms, too, not just the obvious ones (pain, headache).

The limitations of our study are the moderate sample size and the relatively short timeframe. However, the promising outcomes obtained in this research provide a strong rationale for expanding the sample size and the duration of future investigations. Replicating this study on larger samples may confirm the beneficial results of the combined approach and explore the potential of using it on various populations.

## CONCLUSION

Our study contributes to a better understanding of the links between primary headaches, bruxism, and psychosocial issues to improve patients` rehabilitation through a multidisciplinary approach. Furthermore, we aimed to demonstrate the benefits of MT and its combination with counseling and stress relief techniques and strategies. After comparing the results of our patients with their initial assessment scores, our findings after the three-month follow-up period underscore the effectiveness of manual therapy in significantly reducing pain scores. Although the differences between the results achieved through MT and MT+C were not significant at the three-month follow-up, patients who received manual therapy combined with psychological counseling, sleep hygiene counseling, and relaxation techniques related to pain relief had lower frequency and complexity of symptoms, lower functional disability, increased vitality, and an overall increase in the quality of their lives. MT effectively reduced pain and positively affected the biomechanics of the cervical spine by releasing muscular tension, leading to functional improvement. The significant economic burden associated with the costs of TTH, including decreased work performance and increased sick leave, highlights the need for a cost-effective treatment. Manual therapy intervention seems to be a good choice for future cost-effectiveness studies. Additionally, we strongly advise considering the influence of stress and anxiety when managing headache patients.

## Data Availability

The datasets used and analyzed during the current study are available from the corresponding author upon reasonable request.
